# Antiproliferative and apoptotic effect of LY2090314, a GSK-3 inhibitor, in neuroblastoma in vitro

**DOI:** 10.1186/s12885-018-4474-7

**Published:** 2018-05-11

**Authors:** Selvi Kunnimalaiyaan, Victoriana K. Schwartz, Iris Alao Jackson, T. Clark Gamblin, Muthusamy Kunnimalaiyaan

**Affiliations:** Division of Surgical Oncology, Department of Surgery, Medical College of Wisconsin, C4763, Translational and Biomedical Research Center, 8701 Watertown Plank Road, Milwaukee, WI 53226 USA

**Keywords:** GSK-3 inhibitor, Neuroblastoma, GSK-3 alpha, GSK-3 beta, Apoptosis

## Abstract

**Background:**

Neuroblastoma (NB) is a devastating disease. Despite recent advances in the treatment of NB, about 60% of high-risk NB will have relapse and therefore long-term event free survival is very minimal. We have reported that targeting glycogen synthase kinase-3 (GSK-3) may be a potential strategy to treat NB. Consequently, investigating LY2090314, a clinically relevant GSK-3 inhibitor, on NB cellular proliferation and may be beneficial for NB treatment.

**Methods:**

The effect of LY2090314 was compared with a previously studied GSK-3 inhibitor, Tideglusib. Colorimetric, clonogenic, and live-cell image confluency assays were used to study the proliferative effect of LY2090314 on NB cell lines (NGP, SK-N-AS, and SH-SY-5Y). Western blotting and caspase glo assay were performed to determine the mechanistic function of LY2090314 in NB cell lines.

**Results:**

LY2090314 treatment exhibited significant growth reduction starting at a 20 nM concentration in NGP, SK-N-AS, and SH-SY-5Y cells. Western blot analysis indicated that growth suppression was due to apoptosis as evidenced by an increase in pro-apoptotic markers cleaved PARP and cleaved caspase-3 and a reduction in the anti-apoptotic protein, survivin. Further, treatment significantly reduced the level of cyclin D1, a key regulatory protein of the cell cycle and apoptosis. Functionally, this was confirmed by an increase in caspase activity. LY2090314 treatment reduced the expression levels of phosphorylated GSK-3 proteins and increased the stability of β-catenin in these cells.

**Conclusions:**

LY2090314 effectively reduces growth of both human MYCN amplified and non-amplified NB cell lines in vitro. To our knowledge, this is the first study to look at the effect of LY2090314 in NB cell lines. These results indicate that GSK-3 may be a therapeutic target for NB and provide rationale for further preclinical analysis using LY2090314.

## Background

Neuroblastoma (NB) is the most common extracranial solid tumor found in children with over 650 cases diagnosed each year in the United States and it is responsible for 15% of pediatric cancer deaths [1]. The average age of diagnosis is 19 months and over 90% of cases are diagnosed at age 5 or younger. Developed from aberrant primordial neural crest cells during embryogenesis, this cancer type manifests in a variety of clinical presentations as it springs forth from the widespread tissue of the sympathetic nervous system in particular. Most primary tumors are found in the abdomen and more commonly, the adrenal medulla; however, other sites such as the thorax, pelvic region, and neck can be involved [2]. Timely diagnosis and accurate staging is critical in the management and prognosis of NB [3]. Children can be categorized mainly as low, intermediate, or high-risk. Low and intermediate risk patients have high success rates of cure (95 and 90% respectively), with the majority of children effectively treated by surgery and if necessary, available chemotherapy regimens [4]. However, risk severity increases and survival rates decrease significantly depending on tumor characteristics which indicate amplification of MYCN, an oncogene shown to be correlated with poor prognosis [5]. High-risk NB patients are defined by having either metastatic disease diagnosed at an advanced age (> 18 months) or MYCN amplification in tumor. Chemotherapy, surgical resection, hematopoietic stem cell transplant, and immunotherapy encompass the standard treatment regimen available for high risk patients today, however, survival rates remain low and relapse rates high [2, 4, 6]. Unfortunately, chemotherapy resistance remains a critical problem in NB treatment, however, multi-drug regimens and novel therapies have been developed in recent years to combat this issue but targeted treatment is still needed [2, 6–8].

Several intracellular signaling pathways have been demonstrated to play a key role in embryonal tumor biology, including growth factors controlling tumor proliferation, survival, differentiation and metastasis [9–14]. Furthermore, glycogen synthase kinase-3 (GSK-3) is a positive regulator of cancer cell proliferation and survival in advanced cancer and thus is considered a viable therapeutic target for the treatment of a broad spectrum of cancers [15–18]. GSK-3 is considered an oncogene and is highly expressed in NB [19–22]. Several GSK-3 inhibitors have been evaluated in vitro and in vivo, however many of them have not been tested in humans via clinical trials. Earlier, we have reported that the thiazole AR-A014418 reduced neuroblastoma growth and associated with reduction in active phosphorylation of GSK-3 α [19]. Recently, Tideglusib has been shown to induce apoptosis in human NB cells [23]. Though a number of small molecular GSK-3 inhibitors have been studied both in vitro and in vivo animal models, the majority of them are not advanced as drug candidates into the clinic. Recently, LY2090314 has been shown to be a potent, selective inhibitor of GSK-3 and, initial safety studies were conducted in a first-in-human, phase I dose escalation study evaluating the treatment efficacy in patients with advanced solid tumors [24]. To date Tideglusib and LY2090314 have been shown to be well tolerated in clinic [18]. However, the effect of LY2090314 on NB specifically is not known. Therefore, we hypothesize that LY2090314, a clinically relevant GSK-3 inhibitor, will effectively inhibit neuroblastoma cellular proliferation. In this study, we show that LY2090314 significantly inhibits cellular proliferation of three different human NB cell lines and this is associated with reduced GSK-3 phosphorylation. Furthermore, we show that the reduction in growth is due to apoptosis.

## Methods

### Cell lines and culture conditions

Human NB cell lines SK-N-AS (CRL-2137) and SH-SY-5Y (CRL-2266) were purchased from American Type Culture Collection (ATCC, Rockville, MD, USA) and NGP cells were a kind gift from Dr. Thiele at the National Cancer Institute (Bethesda, MD, USA) and were authenticated before receipt and use. NGP, SH-SY-5Y, and SK-N-AS cell lines were maintained in RPMI1640 medium (Gibco-BRL, Grand Island, NY, USA), supplemented with 10% fetal bovine serum (Sigma Cell Culture, St. Louis, MO, USA), and a combination of 100 IU/mL penicillin and 100 μg/mL streptomycin (Gibco-BRL, Grand Island, NY, USA) in a humidified atmosphere of 5% CO_2_ in air at 37 °C. LY2090314 and Tideglusib (Selleckchem.com, Houston, TX, USA) were dissolved in dimethyl sulfoxide (DMSO; Sigma-Aldrich). An equivalent amount of DMSO alone in the treatment group served as a control.

### Cell proliferation assays

#### MTT assay

Cell viability of each cell line was measured using a 3-(4, 5-dimethylthiazol-2-yl)-2, 5-diphenyltetrazolium bromide colorimetric (MTT) assay. A 96-well plate was seeded with each cell line and incubated overnight for adhesion. Tideglusib treatment in increasing micromolar concentrations (0 – 30 μM) was added to each well, and for drug comparison, LY2090314, was added separately in increasing nanomolar concentrations, (0 – 1000 nM). Control wells were treated with DMSO. All wells were made in quadruplicate, and all plates were incubated for 48, 72, or 96 h. Cell viability was measured as described [25, 26]. The assay was repeated at least three times.

#### Colony forming unit (CFU) assay

Clonogenic (CFU) assays were performed to determine the ability of NB cells to form colonies after treatment with LY2090314 using the standard protocol as described previously [25, 26]. Briefly, 2000 cells were plated in each well of 6-well plates. Next day, cells were treated with LY209314 and incubated for about 2-3 weeks. Cell colonies were stained with crystal violet and imaged.

#### IncuCyte cell proliferation/confluence assay

IncuCyte Live-cell Imaging system (Essen Bioscience) was used to measure cellular proliferation/confluence of NB cell lines as described previously [25, 26]. Approximately 1000 NB cells were plated onto a 96-well plate and treated with varying concentrations of LY2090314 (0-1000 nM) and incubated in an XL-3 incubator maintained at 37 °C for up to 4 days. During this incubation period, the cells were photographed every 3 h. Using IncuCyte 2011A software, cell confluency was calculated and expressed as an increase in percentage of confluence as compared to control.

### Western blot analysis

Cell lysates were prepared and quantified as described previously [25, 26]. Briefly, cells were lysed with radioimmunoprecipitation assay buffer (RIPA; Thermo Fisher Scientific. Waltham, MA) after treatment with LY2090314. Protein quantification was measured by a BCA (bicinchoninic acid) protein assay (Thermo Fisher Scientific) and thirty micrograms of protein were loaded on to 7.5, 10%, or 12% sodium dodecyl sulfate polyacrylamide gels (Bio-Rad Laboratories). Then proteins were transferred onto a nitrocellulose membrane using the Trans-Blot Turbo Transfer System (Bio-Rad). Membranes were blocked in a 5% dry milk solution and incubated in primary antibody overnight at 4 °C. The following primary antibodies and their dilution ratios were used: survivin (1:500), cyclin D1 (1:500), phosphorylated AKT (1:1000), poly-ADP ribose polymerase (PARP) (1:2000), glyceraldehyde 3-phosphate dehydrogenase (GAPDH) (1:4000), Bcl-2, Mcl-1, β-actin, GSK-3 (all 1:500; Santa Cruz Biotechnologies, Dallas, TX), cleaved PARP, cleaved caspase-3, phospho-GSK-3 ^S9 and S21^ (all 1:1000; Cell Signaling Technology, Boston, MA), and active GSK-3^Y279 and Y216^ (1:1000; Abcam, Cambridge, MA). Next day, the membranes were washed three times in a 1× phosphate buffered solution with .05% Tween-20 buffer and incubated with horse-radish peroxidase linked anti-mouse or anti-rabbit secondary antibody (1:1000; Santa Cruz) depending on the source of the primary antibody. The membranes were developed using Supersignal West Dura or West Femto (Thermo Fisher Scientific) and imaged using the Molecular Images Chemi-Doc XRS^+^ imager (Bio-Rad).

#### Densitometry analysis

After visualizing proteins bands on the membrane, the band intensities were quantified using Image Lab software version 5.2.1, Molecular Images Chemi-Doc XRS^+^ imager (Bio-Rad). The relative number for the band intensities were calculated by normalized against GAPDH.

### Caspase-3 and -7 activities

A Caspase-Glo 3/7 Assay (Promega, Madison, WI) kit was used to measure cleaved caspase-3 and -7 activities from cell lysates after LY2090314 treatment as described previously [25, 26]. Briefly, cell lysates were incubated with caspase-Glo reagents in 96-well plates for 30 min and the luminescence was measured using Infinite M200PRO Microplate reader (TECAN).

### Statistical analysis

One-way ANOVA analysis was performed using a statistical analysis software package (IBM SPSS Statistics version 22). A *p* value of < 0.05 was considered significant. Data were represented as ± SE.

## Results

### LY2090314 inhibits neuroblastoma proliferation, colony formation, and cell confluency

**Fig. 1:**
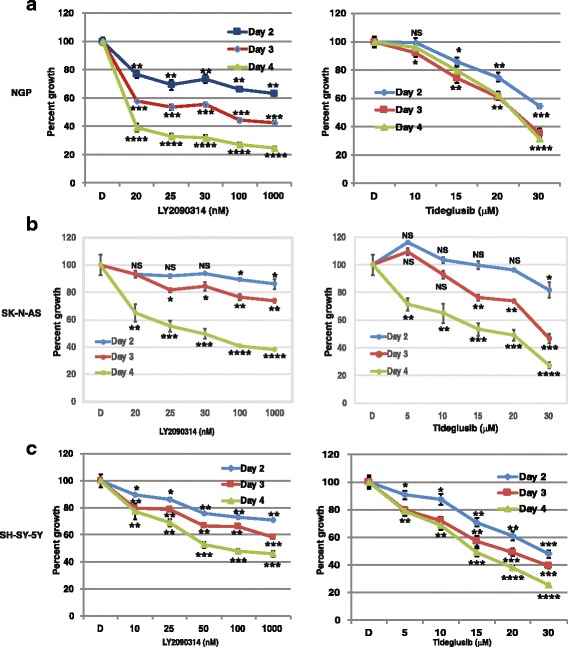
Growth inhibition of human NGP, SK-N-AS, SH-SY-5Y cell lines treated with LY2090314 or Tideglusib by MTT assay. NB cells **a** NGP **b** SK-N-AS and **c** SH-SY-5Y were plated in a 96 well plate and treated with LY2090314 or Tideglusib in increasing concentrations and assay data was collected at 48, 72, and 96 h. Percentage of cell growth seen decreasing in dose and time dependent manner. Tideglusib required μM concentrations compared to LY2090314 (nM) for similar growth reduction. Statistical analysis showed significant growth reduction with increasing concentrations of these drugs. NS, not significant; *, *p* = 0.05; ** *P* < 0.01; ***, *p* > 0.001; ****, *p* > 0.0001. Each experiment was repeated at least three times and the average of these experiments is shown here

**Fig. 2:**
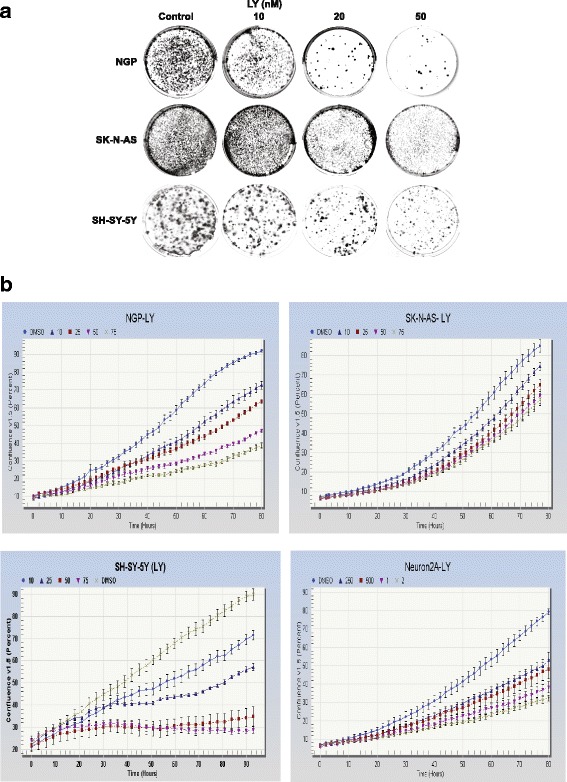
LY2090314 treatment reduced the ability of forming colonies and also decreased the cell confluency in NB cell lines. **a** Colony Forming Unit (CFU) assay performed using NGP, SK-N-AS, and SH-SY-5Y cells, and increasing concentrations of LY2090314 compared to DMSO control. Inhibition by LY2090314 caused a decrease in NB cells ability to form colonies. **b** NGP, SK-N-AS, SH-SY-5Y cells treated with LY2090314 and cell proliferation were monitored in real time. The cells were imaged and the cell confluency was calculated using IncuCyte 2011A software. The changes in cell confluence are used as surrogate markers of cellular proliferation. As seen in this figure, LY2090314 effectively inhibited cell growth by decreasing cell confluency. Experiments were repeated three times and the best are shown here

Several assays and imaging techniques were utilized to determine cellular growth patterns of 3 NB cell lines (NGP, SK-N-AS, and SH-SY-5Y) treated with LY2090314 or Tideglusib. Cells were plated and treated with LY2090314 in increasing nanomolar concentrations (20 nM, − 1000 nM), and proliferation was recorded using a colorimetric, MTT assay at 48 h, 72 h, and 96 h (Fig. 1). For drug comparison, similarly, plates were treated with Tideglusib; however, in increasing micromolar concentrations (10 μM - 30 μM) as demonstrated by T. L. Mathuram et al. [12], and cell proliferation data was recorded. In Fig. 1a, a steep reduction on average of 23% at 48 h, 42% at 72 h, and 61% at 96 h was noted in NGP cells treated with 20 nM of LY2090314. At higher concentrations of 25 nM – 1000 nM LY2090314 in the same cells, there was a more gradual reduction in cell growth, whereas, at 1000 nM a 37% reduction was seen at 48 h, 57% at 72 h, and 75% at 96 h. In comparison, Tideglusib-treated NGP cells at 10 μM decreased by 1, 4, and 8%, at 48, 72, and 96 h respectively. A more significant reduction in proliferation was seen at higher concentrations of 15 μM – 30 μM, where growth decreased 14 - 45% at 48 h, 26 - 65% at 72 h, and 20 - 63% at 96 h. Additionally, a substantial decrease of 22 - 61% can be seen with the much lower concentrations of 20 nM of LY2090314 at 96 h in NGP, SK-N-AS, and SH-SY-5Y cells, whereas, Tideglusib in the lowest micromolar concentration of 10 μM produced a 4 - 50% reduction at 96 h. SK-N-AS and SH-SY-5Y both showed similar decreases in growth, and like NGP, lower concentrations of LY2090314 in the nanomolar range more significantly inhibited growth compared to the micromolar range of Tideglusib. In summary, MTT assay data showed a significant decrease in cellular proliferation in all 3 cell lines treated with LY2090314 at concentrations of 20, 25, 50, 100, and 1000 nM during 48, 72, and 96 h. To confirm MTT results, CFU assays were performed in all cell lines with increasing concentrations of LY2090314 (10 nM – 50 nM) which showed a reduction in NB cells ability to form colonies (Fig. 2a). Lastly, to examine confluency of cells, Incucyte imaging data was collected every 3 h up to 4 days and graphed (Fig. 2b). Decreasing confluency over time is noted in all cell lines treated with increasing concentrations of LY2090314.

### LY2090314 inhibits cell growth via an apoptotic mechanism

**Fig. 3:**
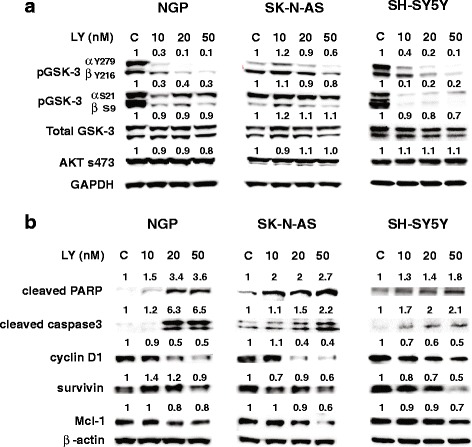
LY2090314 decreased GSK-3 phosphorylation and attenuate apoptosis inhibitor expression. **a** Western blots of all 3 cell lines (NGP, SK-N-AS, SH-SY-5Y), showing decreased phosphorylation of GSK-3α at Tyr279 compared to GSK-3β phosphorylation at Tyr216 as well as decrease in inactive phosphorylation ser 9 and 21. However, there is minimal to no decrease in phosphorylation of active Akt at ser 473. **b** Western blot analysis showed there is an increase in pro-apoptotic markers such as cleaved PARP and cleaved caspase-3, and a decrease in anti-apoptotic markers, cyclin D1, Mcl-1, and survivin. GAPDH was used as loading control. Experiments were repeated at least three times and the best pictures are shown here

**Fig. 4:**
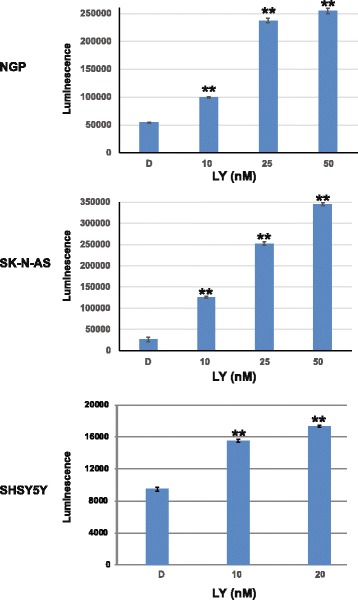
Caspase-3 and-7 activities were measured by Caspase glo assay and showed a dose dependent significant increase of luminescence in all three cell lines treated with LY2090314, indicating increased apoptotic activity. **, *P* > 0.001. Experiments were repeated three times in triplicates and “the averages are shown here”

To determine the pathway mechanism behind the reduction in cell growth, western blotting was performed, proteins of interest were sorted, and the band intensities were measured compared to GAPDH control (Fig. 3). LY2090314, a GSK-3 inhibitor, effectively reduced the protein expression of both the active form of phosphorylated-GSK-3 (αY279, βY216) (70 to 90% reduction with increasing concentrations) and inactive form of phosphorylated-GSK-3 (α S21, β S9) (at least 70% reduction) in NGP and SH-SY-5Y cell lines, potentially indicating GSK-3’s role in inhibiting apoptosis (Fig. 3a). However, SK-N-AS showed 40 and 20% reduction in both active and inactive GSK-3 phosphorylation at higher concentration. Total GSK-3 did not undergo reduction in protein expression in NGP and SK-N-AS cells, whereas slight reduction in SH-SY-5Y cells, indicating that LY2090314 does not have an effect on overall GSK-3 protein synthesis. We did not see a significant change in AKT phosphorylated at serine 473, suggesting that LY2090314 is specific to GSK-3 (Fig. 3a). These cell lines have different genetic groups; SH-SY-5Y (Non-amplified MYCN or single copy, TP53 WT, ALK mutation (F1174 L)), NGP (1p alteration (t(1p), MYCN amplified, ALK wild type, TP53 mutated-MDM2 amplified), and SK-N-AS (1p deletion, MYCN single copy, TP53 mutation (H168R), and ALK WT) and may cause variation in protein expression after LY2090314 treatment. Cleaved PARP and cleaved caspase-3 levels were increased at 10, 20, and 50 nM LY2090314 treatment compared to the control, indicating that the mechanism of cell reduction is apoptosis. Image analysis showed at least 2-fold increase in expression of cleaved PARP and caspase proteins with treatment (Fig. 3b). Furthermore, the anti-apoptotic markers, survivin, and cell survival protein Mcl-1 were also decreased with increasing concentrations. In addition, there was a significant reduction (50%) in cyclin D1 that was seen with increasing concentrations of LY2090314 treatment. Collectively, LY2090314 treatment reduced growth by the induction of apoptosis. This data was confirmed by an increase in capsase-3/7 activity with LY2090314 treatment (Fig. 4).

## Discussion

Neuroblastoma is a rare developmental cancer of the sympathetic nervous system found primarily in infants and young children under 5. Its unique presentation and tumor biology present challenges for effective treatment regimens, notably in patients diagnosed as high risk (>MYCN expression) [6, 27, 28]. In recent years, research has focused on inhibition of the GKS-3 signaling pathway in cancers [16–18, 29]. Studies exploring GSK-3 inhibitors in cancer treatment have been published by us and others within the last several years; however, only Tideglusib and AR-A011418 as a selective GSK-3 inhibitor in neuroblastoma can be found [19, 23]. We have reported that treatment of neuroblastoma cell lines with AR-A014418 reduced the growth and associated with reduction in the level of GSK-3α phosphorylation at Tyr279 compared to GSK-3β phosphorylation at Tyr219. Recently, Tideglusib, another GSK3 inhibitor, has been shown to reduce growth in vitro in neuroblastoma IMR32 cells. Despite this, clinical trials for the GSK-3 inhibitors are very limited. This may be due to the non-specific nature and extensive side effects of general GSK-3 inhibitors. However, the toxicity profile of LY2090314, a selective GSK-3 inhibitor, in combination with carboplatin was reported as similar to the reported cisplatin treatment alone [24]. In this study, we tested 3 different human neuroblastoma cell lines NGP, SK-N-AS, and SH-SY-5Y. These cell lines have different genetic groups; SH-SY-5Y (Non-amplified MYCN or single copy, TP53 WT, ALK mutation (F1174 L)), NGP 1p alteration (t(1p), MYCN amplified, ALK wild type, TP53 mutated-MDM2 amplified), and SK-N-AS (1p deletion, MYCN single copy, TP53 mutation (H168R), and ALK WT). These cell lines showed a clear trend in declining cellular growth in increasing concentrations of LY2090314 as well as a continuous decrease of growth found at longer incubation periods (48, 72, and 96 h). In summary, we found that LY2090314 was highly effective at much lower concentrations in the nanomolar range compared to the micromolar range needed for similar reduction using Tideglusib. Tideglusib was demonstrated to have an anti-proliferative effect in neuroblastoma cell line IMR32 but has not been studied in other NB cell lines [22, 23]. Mathuram et al. used lithium as standard GSK-3 inhibitor to compare the efficiency of growth inhibition by Tideglusib [23]. Therefore, we compared LY2090314 with the Tideglusib, both used in clinics. Our findings indicated that LY2090314 is more potent at nM concentration as an anticancer agent for neuroblastoma, suggesting that it may be used in clinic to treat patients with neuroblastoma disease. Mechanistically, consistent with our prior findings, LY2090314 reduced the level of GSK-3α phosphorylation at Tyr279 compared to GSK-3β phosphorylation at Tyr219 [19]. In addition, inactive phosphorylation of both isoforms (ser21 for α and ser 9 for β) is significantly reduced in SH-SY-5Y cells compared to NGP and SK-N-AS cells. This may be due to cell line specificity. Akt is upstream of GSK-3 and showed minimal to no effect on its phosphorylation, suggesting that LY2090314 is more specific to GSK-3 protein. Furthermore, LY2090314 treatment induced apoptosis, as evidenced by increase in cleaved caspase-3, cleaved PARP and reduced cyclinD1 and possibly survivin and Mcl-1. The mechanism by which GSK-3 acts on cyclin D1 degradation is still controversial [30, 31]. As toxicity remains a common problem with chemotherapy treatments, our results indicate LY2090314 might be a useful alternative to other potential drugs. Our findings thus demonstrate that further preclinical studies are needed to explore the efficacy of LY2090314 in NB. Further, LY2090314 effectively reduces growth of both human MYCN amplified and non-amplified NB cell lines in vitro. To our knowledge, this is the first study completed looking at the effect of LY2090314 in NB cell lines in vitro. In summary, effective inhibition of NB cell growth and contribution of LY2090314 to the clinical benefit observed in other cancer types indicated potential use of LY2090314 is predicted for patients with NB in future.

## Conclusions

Our results confirm that inhibition of GSK-3 by LY2090314 results in NB growth suppression. Importantly, low concentrations of LY2090314 significantly reduced NB cellular growth compared to Tideglusib, another GSK-3 inhibitor. This is the first study in NB, and LY2090314 is a potential agent for the treatment of NB in future.

## Abbreviations

ATCC: American Type Culture Collection
BCA: Bicinchonic acid
GSK-3: Glycogen Synthase Kinase-3
MTT: 3-(4, 5-dimethylthiazol-2-yl)-2, 5-diphenyltetrazolium bromide
NB: Neuroblastoma
PARP: Poly (ADP) ribose polymerase
PBS: Phosphate buffered saline
